# Toll-like receptor and antimicrobial peptide expression in the bovine endometrium

**DOI:** 10.1186/1477-7827-6-53

**Published:** 2008-11-18

**Authors:** Darren Davies, Kieran G Meade, Shan Herath, P David Eckersall, Deyarina Gonzalez, John O White, R Steven Conlan, Cliona O'Farrelly, I Martin Sheldon

**Affiliations:** 1 Department of Veterinary Clinical Sciences, Royal Veterinary College, Royal College Street, London, NW1 0TU, UK; 2 Comparative Immunology Group, School of Biochemistry and Immunology, Trinity College Dublin, Dublin, Ireland; 3 Institute of Comparative Medicine, Faculty of Veterinary Medicine, University of Glasgow, Bearsden Rd, Glasgow, G61 1QH, UK; 4 Institute of Life Science, School of Medicine, Swansea University, Swansea, SA2 8PP, UK

## Abstract

**Background:**

The endometrium is commonly infected with bacteria leading to severe disease of the uterus in cattle and humans. The endometrial epithelium is the first line of defence for this mucosal surface against bacteria and Toll-like receptors (TLRs) are a critical component of the innate immune system for detection of pathogen associated molecular patterns (PAMPs). Antimicrobial peptides, acute phase proteins and Mucin-1 (MUC-1) also provide non-specific defences against microbes on mucosal surfaces. The present study examined the expression of innate immune defences in the bovine endometrium and tested the hypothesis that endometrial epithelial cells express functional receptors of the TLR family and the non-specific effector molecules for defence against bacteria.

**Methods:**

Bovine endometrial tissue and purified populations of primary epithelial and stromal cells were examined using RT-PCR for gene expression of TLRs, antimicrobial peptides and MUC-1. Functional responses were tested by evaluating the secretion of prostaglandin E_2 _and acute phase proteins when cells were treated with bacterial PAMPs such as bacterial lipopolysaccharide (LPS) and lipoproteins.

**Results:**

The endometrium expressed TLRs 1 to 10, whilst purified populations of epithelial cells expressed TLRs 1 to 7 and 9, and stromal cells expressed TLRs 1 to 4, 6, 7, 9 and 10. The TLRs appear to be functional as epithelial cells secreted prostaglandin E_2 _in response to bacterial PAMPs. In addition, the epithelial cells expressed antimicrobial peptides, such as Tracheal and Lingual Antimicrobial Peptides (TAP and LAP) and MUC-1, which were upregulated when the cells were treated with LPS. However, the epithelial cells did not express appreciable amounts of the acute phase proteins haptoglobin or serum amyloid A.

**Conclusion:**

Epithelial cells have an essential role in the orchestration of innate immune defence of the bovine endometrium and are likely to be the key to prevention of endometrial infection with bacteria.

## Background

Microbial infection of the female genital tract is an important cause of disease, infertility and mortality in mammals, particularly cattle and humans. *Bos taurus *is a biologically relevant model to study female genital tract disease because infections are ubiquitous after parturition, often leading to uterine disease. Up to 40% of animals develop clinical metritis within 2 weeks of parturition and endometritis persists for at least 3 weeks in about 20% of cattle [1,2]. The microbes most commonly associated with postpartum uterine disease are *Escherichia coli*, *Arcanobacterium pyogenes *and bovine herpesvirus 4 (BoHV-4) [2-4]. *E. coli *infection paves the way for subsequent infection with *A. pyogenes *and activates BoHV-4 replication [5,6]. The first line of defence against these infections in the female genital tract is the endometrium, which is a mucosa comprising of a layer of single columnar epithelial cells overlying a stroma that contains blood vessels and immune cells as well as endometrial stromal cells. The initial defence of the endometrium against microbes is dependent on innate immune systems, including Toll-like receptors (TLRs), antimicrobial peptides (AMPs), and acute phase proteins (APPs) [7,8]. Furthermore, the immune defence of the endometrium is regulated by the ovarian steroids, oestradiol and progesterone [9]. *Bos taurus *endometrium presents an advantage over human or mouse models for studying innate immunity because it is possible to obtain purified populations of epithelial or stromal cells that are not contaminated by other immune cells [10].

TLRs recognise pathogen associated molecular patterns (PAMPs), and 10 members of the receptor family are widely expressed in the mammalian genome [11]. TLR1, TLR2, and TLR6 recognise bacterial lipids such as lipoteichoic acid (LTA), whereas TLR3, TLR7, TLR8, and TLR9 recognize nucleic acids, often from viruses, although TLR9 also recognises bacterial DNA. TLR4 recognizes lipopolysaccharide (LPS) from Gram-negative bacteria such as *E. coli*, and TLR5 binds flagellin, but the ligand for TLR10 is still not known. The expression of TLRs in the endometrium has been examined in humans [12,13]. However, in cattle, only TLR4 has been studied in detail in endometrial cells, where LPS treatment was characterised by the secretion of prostaglandin E_2 _[14].

AMPs are an ancient component of the immune system, of which β-defensins are the main family, and are particularly important for mucosal immunity [15]. Indeed, the first mammalian β-defensin, Tracheal Antimicrobial Peptide (*TAP*), was identified in cattle. The *Bos taurus *genome sequence has been exploited to expand the AMP family to 57 putative β-defensin genes – the most in any mammal [16]. In a study screening several bovine tissues, the uterus was found to express genes encoding Lingual Antimicrobial Peptide (*LAP*), Bovine neutrophil β-defensins (*BNBD4*, *DEFB5*), and novel bovine β-defensins (*BBD19*, *BBD123 *and *BBD124*) [17]. However, such uterine samples also contain a complex range of cell types, so it is not clear if AMPs are expressed by endometrial cells and have a role in the immune defence of the endometrium.

Acute phase proteins (APPs) such as haptoglobin and serum amyloid A provide non-specific protection against microbes [18]. These APPs are usually produced by the liver, but localised expression has been found in the genital tract of rodents and expression was regulated by the sex steroids [19,20]. Mucin-1 (MUC-1) is an epithelial cell glycosylated transmembrane protein that may also have a role in microbial defence of the endometrium [21]. Although MUC-1 expression has not been evaluated in the bovine uterus, it is expressed by epithelial cells of the human and ovine endometrium [22,23].

Our central paradigm is that the endocrine cells of the endometrium play the key role in the defence of the uterus against microbial infection. The present study tested the hypothesis that endometrial epithelial cells express functional receptors of the TLR family and the non-specific effector molecules for defence against bacteria. The first objective was to establish if bovine endometrium expresses TLRs. The next question was which endometrial cells express functional receptors of the TLR family and the non-specific effector molecules such as AMPs, APPs, and MUC-1 for defence against bacteria.

## Methods

### Tissues and cells

Uteri from postpubertal non-pregnant cattle were collected at a local abattoir immediately after slaughter and kept on ice until further processing in the laboratory. These animals had no evidence of genital disease based on visual inspection and attempted culture of bacteria using standard microbiological techniques. The physiological stage of the reproductive cycle for each genital tract was determined by observation of the ovarian morphology [24]. Tracts with an early corpus luteum (Stage 1) were selected for endometrial tissue and cell culture. Endometrium was carefully dissected free from the underlying tissue and immediately used for cell isolation or put in RNAlater (Qiagen, Crawley, UK) for isolation of mRNA to examine gene expression.

Cell isolation was performed as previously described using only the endometrium from the horn ipsilateral to the corpus luteum [14]. Briefly, tissue was digested in 25 ml sterile filtered digestive solution, which was made by dissolving 50 mg trypsin III (Roche, Lewes, UK), 50 mg collagenase II (Sigma, Poole, UK), 100 mg BSA (Sigma), and 10 mg DNase I (Sigma) in 100 ml phenol-red-free Hanks Balanced Salt Solution (HBSS; Sigma). After a 1.5-h incubation in a shaking water bath at 37°C, the cell suspension was filtered through a 40-μm mesh (Fisher Scientific, Loughborough, UK) to remove undigested material, and the filtrate was resuspended in phenol-red-free HBSS containing 10% fetal bovine serum (FBS; Sigma) and 3 μg/ml trypsin inhibitor (Sigma) (washing medium). The suspension was centrifuged at 100 × *g *for 10 min and, after two further washes in washing medium, the cells were resuspended in RPMI 1640 medium (Sigma) containing 10% fetal bovine serum (PAA laboratories), 50 IU/ml penicillin, 50 μg/ml streptomycin, and 2.5 μg/ml amphotericin B (Sigma). The cells were plated at a density of 1 × 10^5 ^cells in 2 ml per well using 24-well plates (Nunc, Rochester, NY, USA). To obtain separate stromal and epithelial cell populations, the cell suspension was removed 18 h after plating, which allowed selective attachment of stromal cells, and the removed cell suspension was then replated and incubated allowing epithelial cells to adhere. Stromal and epithelial cell populations were distinguished by cell morphology and the purity was greater than 95% as determined by microscopy and the differential production of prostaglandins – stromal cells do not produce prostaglandin F_2α _[14]. The culture media was changed every 48 h until the cells reached confluence. All cultures were maintained at 37°C, 5% CO_2 _in air, in a humidified incubator.

Purified populations of stromal and epithelial cells were collected and stored immediately in RNAlater for mRNA isolation to examine gene expression. To examine if the TLRs were function, 90% confluent stromal and epithelial cells were challenged for 24 h with bacterial PAMPs at concentrations recommended by the manufacture. The PAMPs were 1 μg/ml O55:B5 LPS (Sigma) or 1 μg/ml ultrapurified O111:B4 LPS (InvivoGen, San Diego, CA, USA) for TLR4; 1 μg/ml Pam3CSK4 synthetic bacterial lipoprotein for TLR2 and TLR1 (InvivoGen); 1 μg/ml purified lipoteichoic acid from *Staphylococcus aureus *for TLR2 (LTA, InvivoGen); or, 5 μg/ml LPS-free DNA from *E. coli *for TLR9 (DNA, InvivoGen). The culture supernatants were harvested and frozen at -20°C until used to measure prostaglandin E_2 _as previously described [14]. Briefly, samples were diluted in 0.05 M Tris buffer containing 0.1% gelatin and 0.01% sodium azide. Standards and tritiated tracers for the prostaglandin were purchased from Sigma and Amersham International PLC (Amersham, Little Chalfont, Buckinghamshire, UK), respectively. The antiserum was a generous gift from Prof. N. L. Poyser (University of Edinburgh, Edinburgh, UK), the limit of detection was 2 pg/tube, and intra-assay and inter-assay coefficients of variation were 4.4 and 7.8%, respectively. The effect of the PAMPs on cell survival was evaluated by counting the number of live epithelial and stromal cell using a haemocytometer and the Trypan Blue exclusion method.

To examine AMP, *MUC-1 *and APP expression stromal and epithelial cells were collected and stored immediately in RNAlater for mRNA isolation. To test if these molecules have a role in the endometrial response to bacteria, the cells were also treated for 24 h with 1 μg/ml O55:B5 LPS (Sigma), progesterone (Sigma), or oestradiol (Sigma), as indicated in *Results*.

### Toll-like receptors

Total RNA was isolated with the RNeasy Mini kit (Qiagen) from the samples stored in RNAlater, and DNase treated with RQ1 RNase-free DNase (Promega, Madison, USA). The RNA quality and quantity was determined by spectrophotometry using a NanoDrop-1000 (Labtech Int. Ltd, Ringmer, UK) and 1 μg reverse transcribed using SuperScript II RT (Invitrogen, Paisley, UK) to synthesise first strand cDNA, according to the manufacturers' instructions.

Intron-spanning gene-specific primers for real-time polymerase chain reaction (RT-PCR) were designed for *TLR*s *1 *to *10 *coding sequences published in the National Center for Biotechnology Information database (NCBI, Bethesda, MD, USA) with the aid of Primer3 software [25], and purchased from MWG (Ebersberg, Germany). Details of the PCR primer sequences are provided in Table 1, and each product was sequenced to confirm specificity. Amplification of cDNA was performed using the following conditions: an initial denaturation at 94°C for 5 min; followed by 38 cycles of 94°C for 30 sec, 55–56°C (depending on primer pair T_M_) for 30 sec and 72°C for 30 sec; with a final extension at 72°C for 5 min. Resulting PCR amplicons were separated on 2% agarose gels to confirm the amplification of distinct bands and to assess the expression of each gene.

**Table 1:** Genebank accession numbers, product sizes and nucleotide sequence details of primers for RT-PCR analysis of mRNA expression of bovine *TLR *1 to 10.

Gene	Primer	Sequence 5'>3'	Product Size (bp)	Accession No.
*TLR1*	Sense	ACT TGG AAT TCC TTC TTC ACG A	176	NM_001046504
	Anti-sense	GGA AGA CTG AAC ACA TCA TGG A		
*TLR2*	Sense	GGT TTT AAG GCA GAA TCG TTT G	190	NM_174197
	Anti-sense	AAG GCA CTG GGT TAA ACT GTG T		
*TLR3*	Sense	GAT GTA TCA CCC TGC AAA GAC A	195	NM_001008664
	Anti-sense	TGC ATA TTC AAA CTG CTC TGC T		
*TLR4*	Sense	CTT GCG TAC AGG TTG TTC CTA A	153	NM_174198
	Anti-sense	CTG GGA AGC TGG AGA AGT TAT G		
*TLR5*	Sense	CCT CCT GCT CAG CTT CAA CTA T	172	AY634631
	Anti-sense	TAT CTG ACT TCC ACC CAG GTC T		
*TLR6*	Sense	CCT TGT TTT TCA CCC AAA TAG C	154	NM_001001159
	Anti-sense	TAA GGT TGG TCC TCC AGT GAG T		
*TLR7*	Sense	TCT TGA GGA AAG GGA CTG GTT A	205	DQ333225
	Anti-sense	AAG GGG CTT CTC AAG GAA TAT C		
*TLR8*	Sense	TAA CCT TCG GAA TGT CTC CAG T	232	NM_001033937
	Anti-sense	GTG GGA AAT TCT GTT TCG ACT C		
*TLR9*	Sense	CTG ACA CCT TCA GTC ACC TGA G	156	NM_183081
	Anti-sense	TGG TGG TCT TGG TGA TGT AGT C		
*TLR10*	Sense	ATG GTG CCA TTA TGA ACC CTA C	248	NM_001076918
	Anti-sense	CAC ATG TCC CTC TGG TGT CTA A		

### Antimicrobial peptides

Intron-spanning gene-specific primers for RT-PCR were designed, using the Vector NTI Advance™ software package (Invitrogen, Paisley, UK) and commercially synthesised (Invitrogen) for the following AMPs: *LAP*, *TAP*, *BNBD4*, *DEFB5*, and *BBD119*, *BBD120*, *BBD122*,* BBD122a*,* BBD123*,* BBD124*,* BBD142*, as previously described in detail [17]. The AMPs selected were chosen because they had been identified previously in RNA extracted from a homogenate of uterine tissue [17]. For those gene transcripts identified in the endometrial cells in the present study, quantitative PCR was performed. Each reaction was carried out in duplicate in a total volume of 25 μl with 2 μl of cDNA (20 ng/μl), 12.5 μl 2 × PCR master mix (Stratagene Corp., La Jolla, CA, USA), and 10.5 μl primer/H_2_O. Optimal concentrations of primers were determined by titrating 100, 300 and 900 nM final concentrations of the forward and reverse primers. Real time qRT-PCR was performed using an MX3000P^® ^quantitative PCR system (Stratagene Corp.) with the following cycling parameters: 95°C for 10 min followed by 40 cycles of 95°C for 30 sec, 60°C for 1 min and 72°C for 30 sec followed by amplicon dissociation. Resulting PCR amplicons were separated on 2% agarose gels to confirm the amplification of distinct bands and to assess the expression of each gene. The 2^-ΔΔCt ^method was used to determine median fold changes in gene expression.

### MUC-1

Total RNA was isolated as above, with intermediate on-column DnaseI digestion step (Qiagen, UK) and 1 μg RNA was reverse transcribed using random decamer primers (RETROscript™, Ambion, UK). Gene specific primer pairs were designed (Beacon Design 2.0, Premier Biosoft, USA) for *MUC-1 *(sense 5'-TGTGGTGGTAGAATTAACTC-3'; antisense 5'-ACTAACTCCGCTGATGG-3'; 120 bp) and *β-ACTIN *(sense 5'-ATCGGCAATGAGCGGTTCC-3'; antisense 5'-GTGTTGGCGTAGAGGTCCTTG-3'; 143 bp) as a reference amplicon. Amplification reactions were prepared in a volume of 20 μL by adding 10 μL of SYBR-Green Supermix 2× containing the Thermo-Start^® ^DNA Polymerase (ABgene), 2.5 μL of each primer (4 μM) and 5 μL of serial dilutions of cDNA. RT-PCR amplifications were done in triplicate in 96-well optical reaction plates and run in the BioRad IQ iCycler; genomic DNA and RNA were used as positive and negative controls, respectively. Plates were heated first to 95°C for 15 min to activate the Thermo-Start^® ^DNA Polymerase enzyme and run for 50 cycles of 15 sec at 95°C, 30 sec at the optimal annealing temperature for each primer pair and 30 sec at 72°C, followed by 1 cycle of annealing at 55°C for 30 sec and 1 cycle of denaturation at 95°C for 30 sec. To obtain the melting curves for each sample a final step of 40 cycles was performed for 10s at 53°C, increasing the set point temperature by 1°C per cycle up to a maximum temperature of 94°C. No amplicons were obtained using RNA directly in the PCR reaction. Relative quantification of gene expression data was determined from threshold cycle (Tc) values for each sample. Serial dilutions of cDNA were used to plot a calibration curve, and gene expression levels quantified by plotting Tc values on the curve. Expression levels were normalised with values obtained for the internal reference gene, and fold expression calculated as a ratio of transcript levels between treated and control samples.

### Acute phase proteins

The concentration of haptoglobin was determined using a haptoglobin-haemoglobin binding assay as described previously [26]. The concentrations of serum amyloid were determined using an enzyme linked immunosorbent assay kit (Tridelta Development PLC, Dublin, Ireland) according to the manufacturer's instructions [26]. The limits of detection of the haptoglobin and serum amyloid A assays were 20 μg/ml and 0.33 μg/ml, respectively.

### Analysis

Comparisons between treatment groups were made using ANOVA with Bonferroni Posthoc tests for normally distributed data, and Kruskal-Wallis tests for fold changes in gene expression. Significance was attributed when *P *< 0.05 and data are reported as mean ± SEM.

## Results

### Toll-like receptors

**Figure 1:**
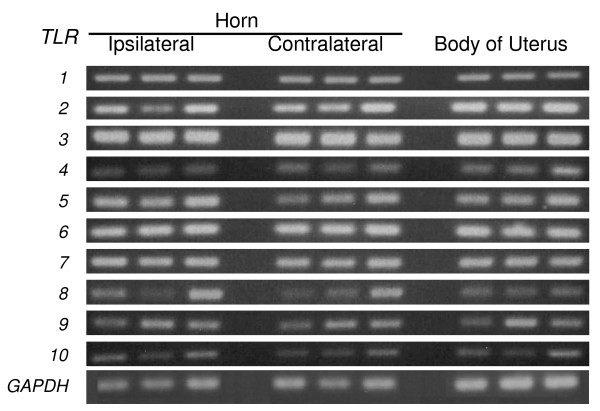
***TLR 1 *to *10 *gene expression by bovine endometrium collected from the uterine body, and the uterine horn ipsilateral or contralateral to the ovary containing the corpus luteum (n = 3 per location).** Also shown is *GAPDH *expression for comparison. RNA was isolated as described, and the resulting cDNA was analyzed by PCR for the presence of *TLR *gene transcripts using the primer pairs described in Table 1.

**Figure 2:**
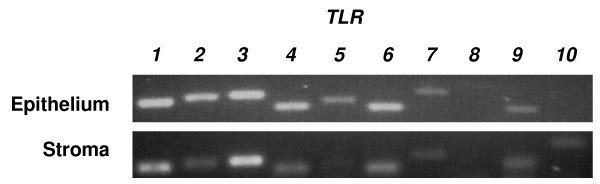
***TLR 1 *to *10 *gene expression by bovine endometrial stromal and epithelial cells.** Also shown is *GAPDH *expression for comparison. RNA was isolated as described, and the resulting cDNA was analyzed by PCR for the presence of *TLR *gene transcripts using the primer pairs described in Table 1. A representative result is shown (n = 3).

Gene transcripts for the ten mammalian *TLR*s were detected in bovine endometrium (Fig. 1). The level of expression did not differ significantly between whole endometrium collected from the body of the uterus, or the horn ipsilateral or contralateral to the corpus luteum. Endometrium contains endothelial, immune and blood cells, so the expression of TLRs was also examined in purified populations of epithelial and stromal cells, which were free of immune cell contamination as determined by the absence of mRNA for the pan-leukocyte marker *CD45 *(data not shown) [14]. Epithelial cells expressed *TLR*s *1 *to *7 *and *9*; stromal cells expressed *TLR*s *1*-*4*, *6*, *7*, *9 *and *10 *(Fig. 2).

**Figure 3:**
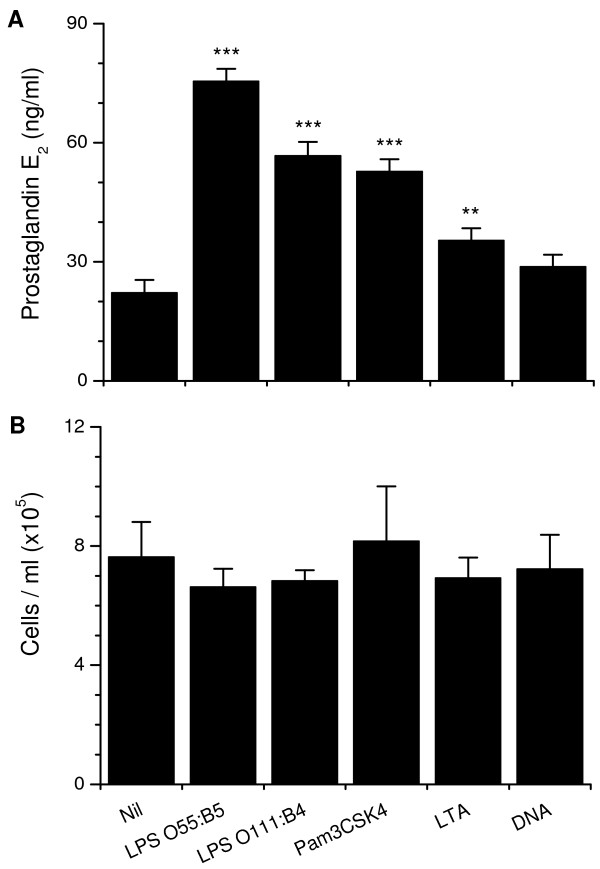
**Endometrial epithelial cell (a) secretion of prostaglandin E_2 _and (b) cells per ml after 24 h treatment with 1 μg/ml O55:B5 *E. coli *LPS, 1 μg/ml ultrapurified O111:B4 *E. coli *LPS, 1 μg/ml Pam3CSK4, 1 μg/ml lipoteichoic acid (LTA) from *Staphylococcus aureus*, or 5 μg/ml LPS-free *E. coli *DNA (n = 6).** Values differ significantly from control, ***P < 0.001, **P < 0.01.

*E. coli *is the first pathogen associated with uterine disease after parturition, inducing prostaglandin E_2 _secretion by epithelial cells [14]. So, a range of purified bacterial PAMPs were used to test if the TLRs associated with detection of bacteria were functional. Epithelial cells secreted more prostaglandin E_2 _than controls when treated with LPS, Pam3CSK4, or LTA, but not bacterial DNA (Fig. 3a). The PAMPs did not affect cell survival (Fig. 3b). The secretion of prostaglandin E_2 _was maximal for the O55:B5 LPS and this was greater than O111:B4 LPS (P < 0.05), so subsequent experiments used O55:B5 *E. coli *LPS as the standard PAMP to challenge cells.

### Antimicrobial peptides

**Figure 4:**
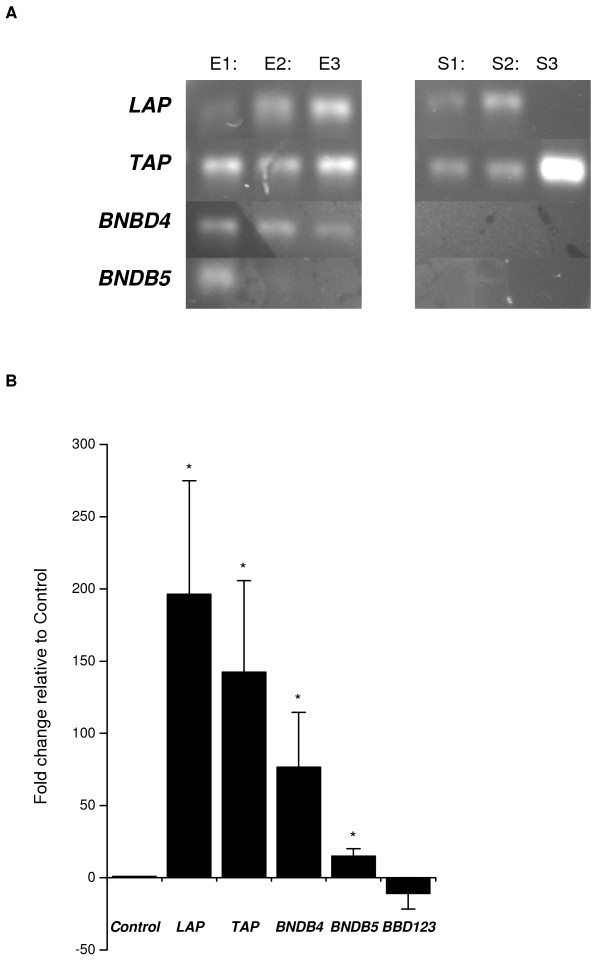
**A analysis of antimicrobial peptide mRNA by epithelial and stromal cells.** RNA was isolated as described, and the resulting cDNA was analyzed by RT-PCR for the presence of *TAP*, *LAP*, *BNBD4 *and *DEFB5 *gene transcripts as described in *Materials and Methods*. A representative result is shown (n = 3 epithelial, E1–3, and stromal samples, S1–3). B analysis of antimicrobial peptide mRNA expression by epithelial cells. Endometrial epithelial cells were stimulated with 1 μg/ml O55:B5 *E. coli *LPS for 24 h and harvested. *TAP*, *LAP*, *BNBD4*, *DEFB5 *and *BBD123 *mRNA was quantified as described in *Materials and Methods*, and the data presented as fold change relative to gene expression in control cells (n = 3) Values differ significantly from control, * P < 0.05.

Gene transcripts for *LAP*, *TAP*, *BNBD4 *and *DEFB5 *(Fig. 4a) and a weak expression of *BBD123 *were identified in epithelial cells by qRT-PCR, but there was no expression of *BBD119*, *BBD120*,* BBD122*,* BBD122a*,* BBD124 *and *BBD142*. The stromal cells expressed *LAP *and *TAP *(Fig. 4a) but did not express *BNBD4*, *DEFB5*, *BBD119*, *BBD120*,* BBD122*, *BBD122a*,* BBD123*,* BBD124 *and *BBD142*.

To test if *LAP*, *TAP*, *BNBD4*, *DEFB5 *or *BBD123 *were likely to be important for the response to bacterial infection, endometrial cells were challenged with LPS for 24 h. Quantitative expression of *LAP*, *TAP*, *BNBD4 *and *DEFB5 *was increased relative to control in epithelial cells treated with LPS (Fig 4b). However, the expression of *LAP*, *TAP*, *BNBD4 *or *DEFB5 *was not significantly changed in epithelial cells treated with LTA. In stromal cells treated with LPS there was no consistent change in AMP gene expression, but LTA reduced *LAP *expression (-2.39 fold relative to control; P < 0.05) and increased *TAP *expression (3.79 fold; P < 0.05). Progesterone (5 ng/ml) did not affect AMP gene expression in epithelial or stromal cells (data not shown).

### Acute phase proteins

The concentrations of haptoglobin were below the detectable limit of the assay and the concentrations of serum amyloid A just at the limit of detection for the test, with no differences in APP concentrations between supernatants from control and LPS treated stromal or epithelial cells.

### MUC-1

**Figure 5:**
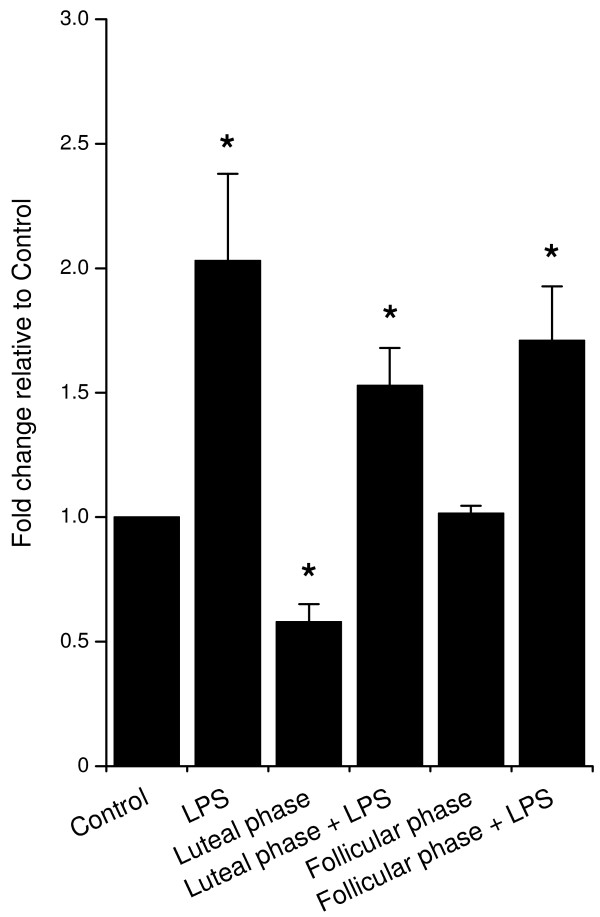
***MUC1 *gene expression by epithelial cells.** Cells were stimulated for 24 h with 1 μg/ml O55:B5 *E. coli *LPS, luteal phase steroid concentrations (5 ng/ml progesterone; 0.3 pg/ml oestradiol) or follicular phase steroid concentrations (0.5 ng/ml progesterone, 3 pg/ml oestradiol) alone or in combination, as indicated. mRNA was quantified as described in *Materials and Methods*, and the data presented as fold change relative to gene expression in control cells (n = 3). Values differ significantly from control, * *P *< 0.05.

Epithelial but not stromal cells expressed *MUC-1 *mRNA, and treatment of epithelial cells with LPS increased the expression of *MUC-1 *(Fig. 5). Luteal phase but not follicular phase concentrations of ovarian steroids reduced *MUC-1 *expression, although neither significantly affected the *MUC-1 *expression in response to treatment with LPS (Fig. 5).

## Discussion

Bacterial infection of the female genital tract is common in cattle particularly after parturition, causing considerable disease, infertility and even mortality [2]. The endometrium is the first line of defence against these bacteria. Key components of innate immunity are the recognition of PAMPs by TLRs, leading to increased expression of AMPs and APPs [11,15]. In the present study endometrial samples expressed *TLR*s 1 to 10, whilst purified populations of epithelial cells expressed *TLR*s *1 *to *7 *and *9*, and stromal cells expressed *TLR*s *1 *to *4, 6, 7, 9 *and *10*. The TLRs appeared to be functional as epithelial cells secreted prostaglandin E_2 _in response to bacterial PAMPs. In addition, the epithelial cells expressed AMPs, such as *TAP *and *LAP*, which were increased when the cells were treated with LPS. Although, there was no evidence of localised secretion of APPs, the epithelial cells also expressed *MUC-1*. It appears that the epithelial cells play a critical role in the innate immune defence of the endometrium against bacteria that cause infertility.

The observation that bovine endometrial tissue expressed gene transcripts for all ten TLRs is similar to human endometrium [27,28]. Human endometrial *TLR *expression is regulated in part by the stage of the cycle [28]. In cattle, there is a progesterone concentration gradient across the uterus with the highest concentration at the tip of the horn ipsilateral to the corpus luteum and the lowest in the contralateral uterine horn [29]. Progesterone concentrations are important because progesterone suppresses immune defences in the bovine endometrium [9]. However, there were no obvious differences in gene transcript expression for any of the *TLR*s between different locations in the uterus.

Endometrial tissue samples contain several cell types including immune cells, so to test the concept that the endocrine cells have a role in immunity, we explored *TLR *expression in purified populations of epithelial and stromal cells, which are free of immune cell contamination in cattle [14]. The epithelial cell expression of *TLR*s *1 *to *7 *and *9*, and stromal cells expression of *TLR*s *1 *to *4, 6, 7, 9 *and *10*, is similar to humans, where expression of *TLR 1 *to *9 *has been reported in endometrial cell lines and in primary uterine epithelial cell cultures [30,31]. The epithelial cell expression of *TLR*s *1, 2, 4, 6 *and *9 *is important because they are required to detect bacterial PAMPs [11]. Bacterial infection is the predominant cause of uterine disease in cattle, and *E. coli *paves the way for other pathogens to cause damage to the endometrium, as well as disrupting endocrine function [5].

Bacterial PAMPs include LPS, LTA and DNA, so we tested the effects of these and the synthetic Pam3CSK4, to gain an insight into whether the cognate TLRs 1, 2, 4, 6 and 9 are functional in the epithelial cells. We chose to evaluate prostaglandin E_2 _concentrations because it is a clear marker of bacterial infection in the bovine endometrium, as well as having an important endocrine role for regulating ovarian cycles and implantation [14,32]. *E. coli *or LPS switches epithelial cell secretion from prostaglandin F_2α _to predominantly prostaglandin E_2_, mediated by the TLR4, MD2, CD14 signalling complex expressed by epithelial cells [14]. The predominant secretion of prostaglandin E_2 _rather than prostaglandin F_2α _is dependent on bacteria or PAMPs stimulating changes in the eicosaniod synthesis pathway, which includes phospholipases and prostaglandin synthases (Herath, unpublished data). This switch in function is important as prostaglandin F_2α _initiates luteolysis whereas prostaglandin E_2 _is luteotrophic in ruminants [33]. Prostaglandin E_2 _is also an important component of the immune response to bacteria and regulates or suppresses inflammation in many tissues [34]. In the present study, LPS, LTA and Pam3CSK4 increased prostaglandin E_2 _secretion and none of the ligands affected epithelial cell survival, extending earlier observations that LPS was detected by human and bovine endometrial cells [14,35]. This stimulation of epithelial cell secretion of prostaglandin E_2 _by several bacterial PAMPs may explain the association between uterine disease and extended luteal phases [36,37]. The secretion of prostaglandin E_2 _was greater for the LPS from O55:B5 than O111:B4 *E. coli*, which may reflect the greater purity of the latter preparation. On the other hand, the O55:B5 LPS provided a potent PAMP to evaluate AMP expression in subsequent experiments.

The AMPs are an important arm of the innate immune defence against bacteria and TLRs mediate their induction in many mamalian tissues [15,38]. In the present study, bovine endometrial epithelial cells expressed several AMPs including *LAP*, *TAP*, *BNBD4 *and *DEFB5*, whilst stromal cells expressed mainly *LAP *and *TAP*. The predominant expression of *TAP *and *LAP *by the epithelial cells is similar to the bovine mammary gland [39]. In humans, β-defensins have also been detected in the endometrium (*HBD1, 2, 3 *and *4*), although there are some differences between studies [40,41]. In the present study, the epithelial cell AMP expression appeared to be of functional importance as the gene expression was increased in epithelial cells treated with LPS. The epithelial cells were consistently more responsive than the stromal cells, and LPS stimulated a greater response than LTA. Similarly in humans, endometrial *HBD2 *mRNA expression is increased after 24 h treatment with LPS, although unlike studies with human cells we did not find that progesterone regulated AMP expression in endometrial cells [40,41]. However, the substantial induction of epithelial *LAP *and *TAP *by LPS treatment probably reflects their defensive role against *E. coli *in cattle, and agrees with the concept that TLRs mediate induction of AMPs in response to PAMPs [15].

The concentrations of APPs, including haptoglobin and serum amyloid A, are substantially increased in the peripheral plasma of cow with bacterial infection of the endometrium [5]. Similar to the AMPs, the APPs such as haptoglobin and serum amyloid A provide non-specific protection against microbes [18]. Although usually produced by the liver, there is evidence of localised APP expression in the genital tract of rodents, regulated by the sex steroids [19,20]. However, in the present study the concentrations of haptoglobin and serum amyloid A proteins were barely detectable in epithelial or stromal cells, and not affected by treatment of cells with LPS. *In vivo *peripheral plasma concentrations are at least 25 times those of the culture supernatants [42]. So, it appears unlikely that localised secretion of APPs plays a major role in endometrial immunity in cattle.

MUC-1 is a glycosylated transmembrane protein commonly expressed by the epithelial cells of mucosal surfaces, including the reproductive tract, gut, testis and mammary gland [21,43]. MUC-1 is expressed in the human and ovine endometrium and has important roles in endometrial receptivity for embryo implantation, and is a marker of endometrial health and fertility in humans [22,23]. However, like AMPs and APPs, MUC-1 also has a role in protecting mucosal surfaces against bacteria. MUC-1 sterically inhibits microbial access to the cell surface and regulates inflammation [21,43]. In the present study, *MUC-1 *expression was detected in the bovine epithelial cells and LPS increased the mRNA expression. In the human endometrium, *MUC-1 *is highly regulated by the ovarian sex steroids [44]. Although luteal phase concentrations of ovarian steroids decreased *MUC-1 *expression, follicular phase concentrations had no effect in the present study. Further, neither steroid combination affected the increase of *MUC-1 *expression stimulated by LPS. This is in contrast to the reduction in LPS-stimulated prostaglandin E_2 _secretion associated with progesterone or oestradiol [14].

## Conclusion

In conclusion, the endometrium is an important first line of defence against invading bacteria that cause disease in cattle and endometrial samples expressed TLRs 1 to 10. The present study explored the concept that the endocrine cells play an important mechanistic role in the defence of the endometrium against bacteria. Purified populations of endometrial epithelial cells expressed *TLR*s *1 *to *7 *and *9*, and stromal cells expressed *TLR*s *1 *to *4, 6, 7, 9 *and *10*. The TLRs appear to be functional as epithelial cells secreted prostaglandin E_2 _in response to bacterial PAMPs. In addition, the epithelial cells expressed AMPs, such as *TAP *and *LAP*, and *MUC-1*, which were upregulated when the cells were treated with LPS. Thus, innate immune defence systems in the epithelial cells of the bovine endometrium are likely to play a key role in the prevention of endometrial infection with bacteria and mediate changes in endocrine function.

## Competing interests

IMS holds research funding under a Department for Environment Food and Rural Affairs (DEFRA) LINK award from Pfizer Animal Health and the Biotechnology and Biological Sciences Research Council (BBSRC; F005121). Darren Davies was supported by a BBSRC CASE Studentship partly funded by Pfizer Animal Health (BBS/S/N/2005/12367). The remaining authors declare that they have no competing interests.

## Authors' contributions

IMS was awarded the grants to fund the work, devised experiments, collated the data and wrote the manuscript. DD performed cell culture work and the molecular biology for TLR analysis. KGM and COF performed the AMP expression work and contributed to the manuscript. SH performed the cell culture PAMP treatments and prostaglandin measurements. PDE performed the acute phase protein measurements. DG, JOW, and RSC completed the *MUC-1 *analysis and contributed to the manuscript. All authors read and approved the final manuscript.
